# Adjuvant chemotherapy—Radiotherapy—Chemotherapy sandwich protocol in resectable soft tissue sarcoma: An updated single-center analysis of 104 cases

**DOI:** 10.1371/journal.pone.0197315

**Published:** 2018-05-22

**Authors:** Christoph Schliemann, Andrea Kerkhoff, Paula Hesse, Sebastian Bröckling, Jendrik Hardes, Arne Streitbürger, Dimosthenis Andreou, Georg Gosheger, Sandra Elges, Eva Wardelmann, Wolfgang Hartmann, Rolf Mesters, Georg Lenz, Normann Willich, Jan Kriz, Hans Eich, Wolfgang E. Berdel, Torsten Kessler

**Affiliations:** 1 University Hospital Muenster, Department of Medicine A, Muenster, Germany; 2 University Hospital Muenster, Department of Orthopedics and Tumororthopedics, Muenster, Germany; 3 University Hospital Muenster, Gerhard-Domagk-Institute for Pathology, Muenster, Germany; 4 University Hospital Muenster, Department of Radiation Oncology, Muenster, Germany; Tata Memorial Centre, INDIA

## Abstract

Adjuvant therapy of local soft tissue sarcomas (STS) after wide surgical excision still is a topic under controversial scientific debate. In this single center report we have offered an adjuvant “sandwich” therapy protocol consisting of 4 cycles of doxorubicin (75 mg/m^2^ i.v. over 1 h on day 1) followed by ifosfamide (5 g/m^2^ i.v. over 24 h starting on day 1) and local radiotherapy scheduled between chemotherapy cycles 2 and 3 to 104 consecutive patients after wide surgical excision (R0) of histologically proven high-grade STS. After a mean follow-up of 39 months (range 5–194 months) relapse free survival (RFS) at 2 and 5 years was 68.1% (95% CI, 58.5–77.7%) and 61.2% (95% CI, 50.4–71.6%). When analyzing the 82 STS cases of the extremities only 2- and 5-year RFS was 74.0% (95% CI, 64.0–84.0%) and 65.3% (95% CI, 53.7–76.9%). By intent-to-treat analysis, the overall survival (OS) at 2 years was 87.3% (95% CI, 80.5–94.1%) and 75.6% (95% CI, 65.2–86.0%) at 5 years, while OS for STS of the extremities only cohort was 90.5% (95% CI, 83.7–97.3%) and 79.0% (95% CI, 68.4–89.6%), respectively. Tolerability of the treatment was good. This analysis demonstrates the feasibility of adjuvant chemoradiotherapy and reflects the results of the long lasting intensive multidisciplinary team approach at our “high-volume” sarcoma center. The long-term survival in our patients is among the highest reported and the low local and distant recurrence rate in high-risk STS is at least comparable to the published data.

## Introduction

Adult soft tissue sarcomas (STS) constitute a very heterogeneous group of malignancies, including more than 70 histological subtypes which can arise from mesenchymal tissue at virtually every site of the body [1]. STS are rare tumors, with an overall incidence of approximately 5 cases per 100.000 per year [2, 3]. Surgical resection is the standard and the only potentially curative therapy for STS [3–5]. However, a large proportion of STS patients eventually develop metastases, particularly those with high-grade histology, large tumors and deep tumor localizations [3, 6–8]. Even though resection of oligometastatic disease may cure a minority of patients, the overall prognosis of disseminated STS remains highly unsatisfactory. Thus, effective treatments to prevent or at least to delay local and distant relapses after surgical resection are urgently needed. However, the heterogeneity and the rarity of STS contribute to a high degree of uncertainty in terms of post-surgery treatment recommendations. In particular, there is still no consensus on the clinical value of adjuvant chemotherapy in STS, although several controlled trials have been published over the last decades and have been pooled into meta-analyses [5, 9, 10]

With the aim to find an optimal compromise between reducing the risk of distant recurrence and preventing local relapse, we have previously investigated the feasibility, toxicity and efficacy of adjuvant radiation therapy ‘‘sandwiched” in the middle of four chemotherapy cycles of doxorubicin and ifosfamide in patients with STS [11]. Here, we report an updated analysis on 104 consecutive STS patients treated according to this protocol at our sarcoma center.

## Material and methods

### Patients

The patients in this study represent an extension cohort treated according to the original protocol of the phase II clinical trial published in 2012 [11]. Briefly, all patients diagnosed with high risk but resectable soft tissue sarcoma (STS) were discussed in our interdisciplinary tumor board between orthopedic surgeons, radiation oncologists, medical oncologists, radiologists and pathologists in order to evaluate multi-modal therapy. All histologies underwent a reevaluation regarding histotype and grading according to French Federation of Cancer Centers Sarcoma Group (FNCLCC) by our experienced sarcoma pathologist and her team for preparation of this manuscript. Only patients who received chemotherapy were considered for this analysis. According to the original protocol patients with clearly resectable tumors were offered to have wide resection first followed by four cycles of chemotherapy with ifosfamide/doxorubicin and local radiation performed between cycles 2 and 3. Informed consent was obtained from all patients after extensive information was given about the nature and extent of disease, available treatment options, effects and side effects of treatment and data management for scientific evaluation. Both the original study protocol and this updated analysis were approved by the local ethics committee (Ethikkommission der Aerztekammer Westfalen-Lippe; 2016-296-f-S).

#### Surgery

Diagnosis of STS was confirmed by histological examination of specimens obtained by preoperative open biopsy performed by an experienced orthopedic surgeon, and in some cases by percutaneous CT-guided biopsy. After initial staging, discussing all results in our interdisciplinary tumor board and obtaining informed consent by the patient, definitive surgery was performed with the intention of complete and wide en bloc tumor resection (R0). Only patients with locally advanced STS in close proximity to non-resectable healthy structures (e.g., neuro-vascular bundles) limiting a wide resection upfront were offered to receive preoperative systemic and/or radiation therapy.

#### Chemotherapy

After ruling out contraindications for systemic chemotherapy with the substances used and obtaining informed consent by the patient the first two cycles were administered. In most cases chemotherapy was started after completion of post-surgery reconvalescence and wound healing within the first 4 weeks. The protocol used consisted of doxorubicin (75 mg/m^2^ i.v. over 1 h on day 1) followed by ifosfamide (5 g/m^2^ i.v. over 24 h starting on day 1), using standard prophylactic measures for anti-emetic prophylaxis. In order to shorten the period of chemotherapy induced leukocytopenia G-CSF was administered interventionally. Chemotherapy was repeated every 21 days. After completion of local radiotherapy, chemotherapy was resumed with cycles 3 and 4, not before 10 days after the last administration of radiotherapy.

#### Radiotherapy

‘‘Sandwiched” between chemotherapy cycles 2 and 3, patients underwent percutaneous radiation therapy as involved-field onto the region where surgery was performed. A total dose of 50.4 Gy fractionated in daily 1.8–2 Gy on 5 days/week were applied onto the operative field. The radiation field was reduced to the initial tumor bed by modern CT-guided radiation planning and a boost was applied to a cumulative dose of 60 Gy.

#### Toxicity and follow-up

Before adjuvant chemoradiation, baseline characteristics were evaluated. During chemotherapy, weekly clinical evaluation as well as blood counts and biochemistry measurements were recorded according to the Common Terminology Criteria for Adverse Events v3.0 (CTCAE). Before, during and after radiotherapy, we recorded radiation-induced acute toxicity (RTOG grade). Regular follow-up visits or telephone interviews were performed.

### Statistical methods

The primary endpoint of this updated analysis was recurrence free survival (RFS) at 2 and 5 years as calculated by the Kaplan-Meier survival analysis in SPSS. Events for RFS included local relapse, distant relapse or death, whatever occurred first. Local and distant RFS was defined as the time between biopsy and recurrence of disease either at the local site or at a distant (metastatic) site, respectively. Secondary endpoints were treatment-related morbidity and overall survival (OS). OS was defined as the time between biopsy and death from any cause. Separate analyses were performed for the overall cohort (n = 104) as well as for those with STS of the extremities only (n = 82). All statistical analyses were performed using the SPSS Software package (version 24).

## Results

### Patient characteristics

Between August 1997 and 2014, 104 patients entered this protocol at our institution and were regularly followed. Baseline characteristics are presented in Table 1. About 80% patients presented with STS of the extremities. These represented a wide array of histologies, with the majority being undifferentiated pleomorphic sarcoma, myxofibrosarcoma and synovial sarcoma. 87/104 patients had high-risk STS with aggressive G3 tumors and 73/104 patients had a UICC stage III.

**Table 1:** Baseline patient characteristics.

Variable	N = 104
**Age (years)**	
**Median (range)**	48 (21–71)
**Gender**	
**Female, n (%)**	49 (47.1)
**Male, n (%)**	55 (52.9)
**Disease site**	
**Extremity, n (%)**	82 (78.9)
**Trunk, n (%)**	19 (18.3)
**Neck, n (%)**	3 (2.9)
**Histology, n (%)**	
**Synovial sarcoma**	17 (16.3)
**Undifferentiated pleomorphic sarcoma**	43 (41.3)
**Leiomyosarcoma**	6 (5.8)
**Liposarcoma**	10 (9.6)
**Myxofibrosarcoma**	11 (10.6)
**Rhabdomyosarcoma**	6 (5.8)
**Other**	11 (10.6)
**Grading**	
**G2, n (%)**	11 (10.6)
**G3, n (%)**	87 (83.7)
**Tumor size**	
**<5 cm, n (%)**	31 (29.8)
**5–10 cm, n (%)**	31 (29.8)
**>10 cm, n (%)**	29 (27.9)
**Unknown, n (%)**	13 (12.5)
**Extent of resection**	
**R0, n (%)**	93 (89.4)
**R1, n (%)**	9 (8.7)
**Unknown, n (%)**	2 (1.9)

### Surgery

All patients underwent definitive surgery. In 93/104 patients wide R0 resection could be achieved, in 9/104 patients the grade of resection was classified as marginally involved (R1) and in 2/104 patients the grade of resection was not documented.

### Adherence to protocol and feasibility

The cumulative ifosfamide and doxorubicin doses are shown in Table 2. When calculated per cycle, the mean administered ifosfamide and doxorubin doses are close to the per-protocol doses of 5 g/m^2^ and 75 mg/m^2^, respectively. Overall, all 104 patients started chemotherapy according to protocol and 94 received four full dosed cycles. In 7 patients one cycle was omitted due to infection or other complications and 3 patients had recurrent disease during adjuvant therapy and were switched to 2^nd^ line regimen.

**Table 2:** Treatment protocol and adherence to protocol.

Variable	N = 104
**Chemotherapy, n (%)**	104 (100)
**4 cycles**	94 (90.1)
**< 4 cycles**	10 (9.6)
**Cumulative ifosfamide dose, mean (g/m**^**2**^**) (stdev)**	19.4 (2.5)
**Cumulative doxorubicin dose, mean (g/m**^**2**^**) (stdev)**	290.2 (37.2)
**Dose reduction, n (%)**	12 (11.7)
**Radiotherapy, n (%)**	89 (86.4)
**Sandwich, n (%)**	79 (76.0)
**Before chemotherapy, n (%)**	10 (9.6)
**Total radiotherapy dose, mean (Gy) (stdev)**	61.7 (4.7)
**Adherence to protocol, n (%)**	
**Yes**	84 (80.8)
**No**	19 (18.3)
**Recurrent disease during treatment**	3 (2.9)
**Infection/complications**	10 (9.6)
**Mesh graft too fresh for radiotherapy**	1 (1.0)
**External previous treatment**	2 (1.9)
**Neoadjuvant treatment**	2 (1.9)
**Patient‘s wish**	1 (1.0)
**Time from surgery to start of cycle 1 of chemotherapy**	
**Median (range), days**	31 (8–148)
**Time from surgery to last chemotherapy (cycle 4)**	
**Median (range), days**	177 (80–271)
**Median follow-up (range), month**	39 (5–194)

Radiotherapy was applied in 89/104 patients, which was “sandwiched” between chemotherapy cycle 3 and 4 in 79 patients, while 10 patients received radiotherapy first in order to maximize local control in cases without R0 resection. The median time span for the whole protocol starting with surgery and ending with the last cycle of chemotherapy was 177 days.

### Chemotherapy, radiotherapy and toxicity

Detailed data for toxicity are displayed in Table 3. Bi-weekly blood counts and biochemistry analysis during the chemotherapy phase revealed that most patients had only minor or moderate hematological toxicity. Grade 3/4 neutropenia occurred in 14 (11 grade 3; 3 grade 4) patients, grade 3 thrombocytopenia in 3 patients, grade 3 anemia in 7 patients.

**Table 3:** Treatment related toxicities according to CTC.

Variable	N = 94
**Anemia**	
**Grade 3, n (%)**	7 (7.4)
**Grade 4, n (%)**	0
**Leukocytopenia**	
**Grade 3, n (%)**	11 (11.7)
**Grade 4, n (%)**	3 (3.2)
**Thrombocytopenia**	
**Grade 3, n (%)**	3 (3.2)
**Grade 4, n (%)**	0
**Nausea and vomiting**	
**Grade 3, n (%)**	4 (4.3)
**Grade 4, n (%)**	0 (0)
**Radiation-related acute skin toxicity**	
**Grade 3, n (%)**	3 (3.2)

The administered cumulative mean dose of ifosfamide and of doxorubicin did not differ significantly from the planned dose, indicating that chemotherapy-related toxicity did not require dose reductions. After radiotherapy and follow-up, patients were examined and interviewed for radiation-related acute toxicity of the skin, joint and bone. Only 3 patients experienced grade 3 toxicity of the skin. No chronic radiation-related toxicity has been recorded so far.

### Outcome

**Fig 1:**
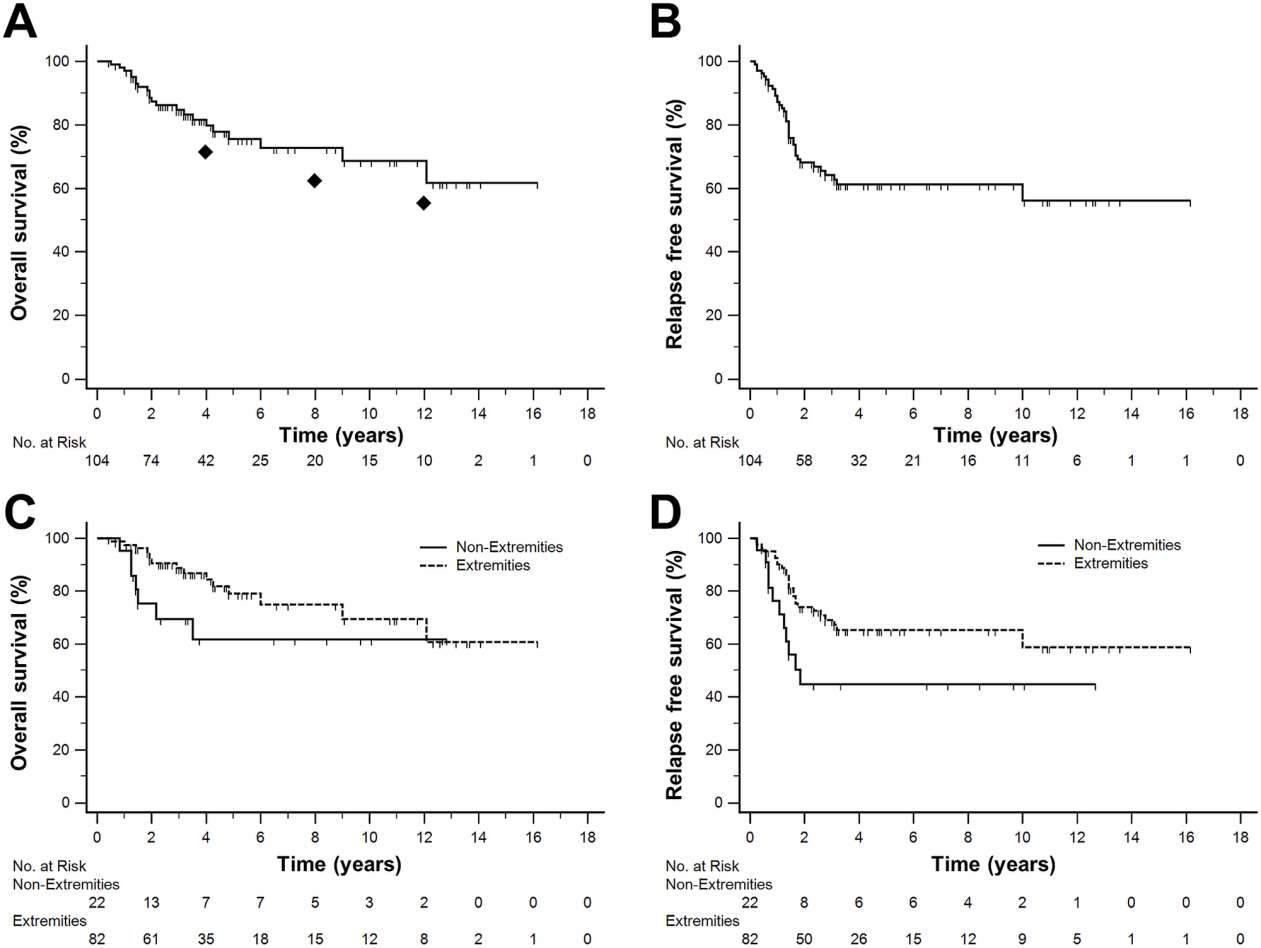
Kaplan-Meier estimates of recurrence free survival (RFS) and overall survival (OS). (A) OS in the entire cohort (n = 104) at 2 years was 87.3% (95% CI, 80.5–94.1%) and 75.6% (95% CI, 65.2–86.0%) at 5 years; ◆ indicates calculated survival probability according to MSKCC nomogram at 4, 8, and 12 years. (B) RFS in the entire cohort at 2 and 5 years was 68.1% (95% CI, 58.5–77.7%) and 61.2% (95% CI, 50.4–71.6%) (C) OS stratified by tumor localization (extremities only cohort 90.5% (95% CI, 83.7–97.3%) and 79.0% (95% CI, 68.4–89.6%) at 2 and 5 years. (D) RFS stratified by tumor localization (extremities only cohort was 74.0% (95% CI, 64.0–84.0%) and 65.3% (95% CI, 53.7–76.9%) at 2 and 5 years.

After a mean follow-up of 39 (range 5–194) months data were analysed for overall RFS and OS, as well as for RFS and OS as stratified by tumor localization (extremities vs. non-extremities). Of the 104 patients treated 67 are free of recurrence and alive and 37 patients had either local or distant relapse. Of those, 12 were successfully treated to obtain a second complete remission, 5 are alive with recurrence and still under therapy and 20 patients died due to relapse (Table 4). RFS at 2 and 5 years was 68.1% (95% CI, 58.5–77.7%) and 61.2% (95% CI, 50.4–71.6%) (Fig 1B). When analyzing the 82 STS cases of the extremities only, 2- and 5-year RFS was 74.0% (95% CI, 64.0–84.0%) and 65.3% (95% CI, 53.7–76.9%) (Fig 1D). (Fig 1B).

**Table 4:** Outcome.

Variable	N = 104
**First complete remission, n (%)**	67 (64.4)
**Recurrent disease, n (%)**	37 (35.6)
**Second complete remission**	12 (11.5)
**After local recurrence**	7 (6.7)
**After distant recurrence**	5 (4.8)
**Death due to recurrent disease**	20 (19.2)
**After local recurrence**	3 (2.9)
**After distant recurrence**	17 (16.4)
**Alive with recurrence**	5 (4.8)
**Death due to other cause, n (%)**	2 (1.9)

**Fig 2:**
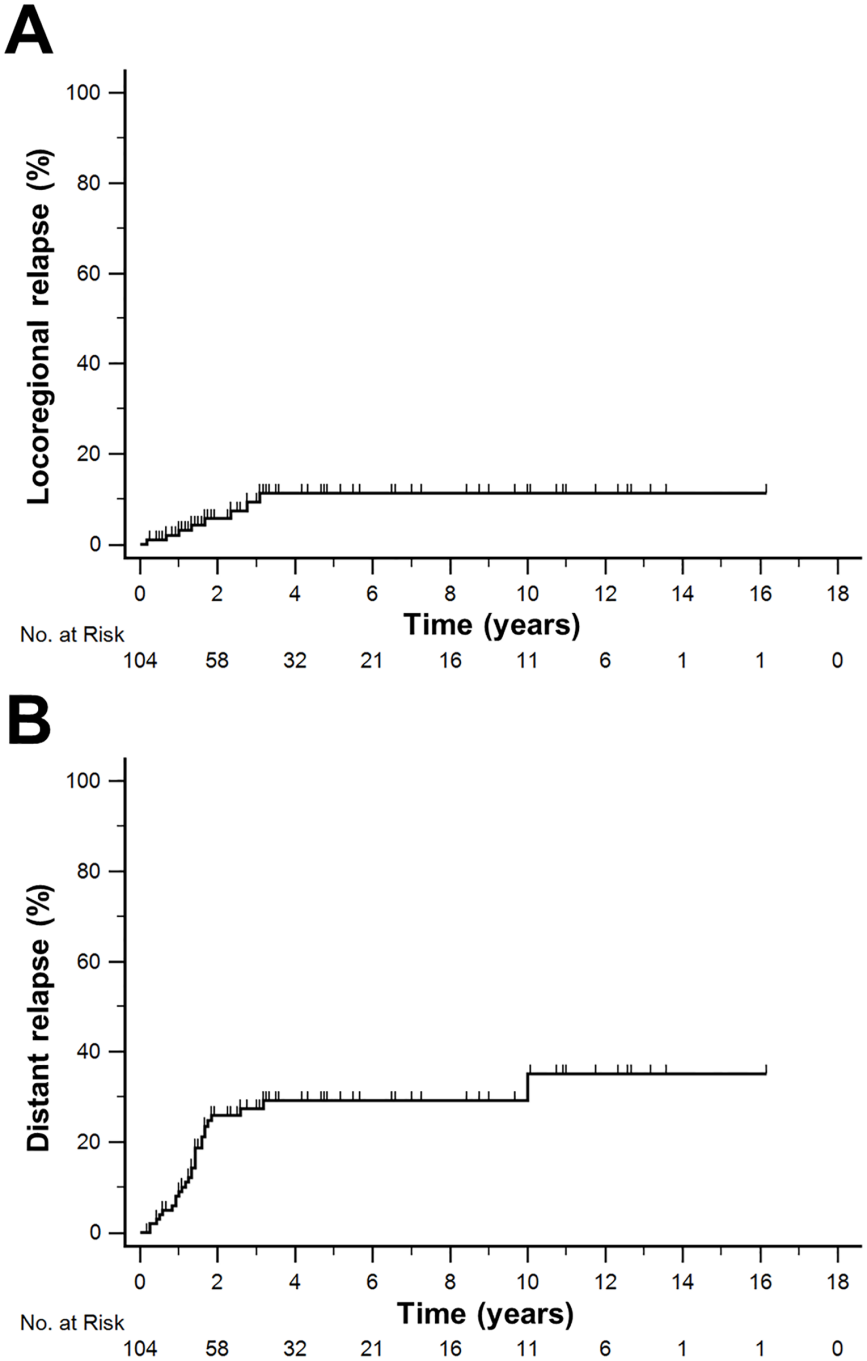
Risk of relapse. (A) Cumulative risk of locoregional relapse: 5.7% (95% CI, 0.7–10.7%) at 2 years and 11.3% (95% CI, 3.5–19.1%) at 5 years. (B) Cumulative risk of distant relapse: 25.9% (95% CI, 16.7–35.1%) at 2 years and 29.2% (95% CI, 19.4–39.0%) at 5 years.

By intent-to-treat analysis, the overall survival (OS) at 2 years was 87.3% (95% CI, 80.5–94.1%) and 75.6% (95% CI, 65.2–86.0%) at 5 years, while OS for STS of the extremities only cohort was 90.5% (95% CI, 83.7–97.3%) and 79.0% (95% CI, 68.4–89.6%), respectively (Fig 1A and 1C). In the entire cohort, the 4-, 8- and 12-year survival predicted by the Memorial Sloan-Kettering Cancer Center (MSKCC) nomogram [12] was 71.5% (95% CI, 68.9–74.1%), 62.5% (95% CI, 59.3–65.7%) and 55.5% (95% CI, 52.0–59.0%) while the observed survival rates at these time points in our cohort were 79.7% (95% CI, 70.5–88.9%), 72.7% (95% CI, 62.3–83.1%) and 68.6% (95% CI, 55.2–82.0%), respectively. We did not observe a significant impact of the whole treatment duration on OS (Cox regression: p = 0.238). Kaplan-Meier estimates for risk of local and distant relapse are presented in Fig 2.

## Discussion

Up to date, two big meta-analyses confirmed the benefits of chemotherapy in high-risk resectable STS with respect to relapse free survival and suggested even a statistically significant benefit in terms of OS for the combination of doxorubicin plus ifosfamide [13, 14]. Pervaiz et al. included 18 randomised trials with over 1900 patients and documented the absolute risk reduction for doxorubicin in combination with ifosfamide in this analysis to be 11% (30% vs. 41% risk of death, p < 0.01). Afonso et al investigated 18 randomized clinical trials (n = 2145) and found an odds ratio for doxorubicin combined with ifosfamide of 0.56 (95% CI, 0.36–0.85; P = .01) in favor of chemotherapy [13]. The Italian Sarcoma Group (ISG) randomized study had demonstrated a survival benefit with five cycles of adjuvant chemotherapy with full-dose epirubicin and ifosfamide in high-risk STS of the extremities versus surgery only [15]. In fact, in this trial there was premature discontinuation of accrual at two years, when a significant difference in the cumulative incidence of distant metastasis was found (45 vs.28%), in favour of the chemotherapy group. The OS was also significantly better in the chemotherapy arm on follow-up at 4 years (69 vs. 50%), but it lost significance at long-term follow-up over 7 years [16].

In contrast, the results of the largest randomized trial (EORTC62931) were rather disappointing [17]. Chemotherapy did not improve relapse-free survival (HR 0.91 [0.67–1.22], p = 0.51) or overall survival (HR 0.94 [0.68–1.31], p = 0.72). This result was confirmed by the pooled analysis of two phase III trials by Le Cesne et al in 2014 [4]. Kasper et al analysed the prognostic factors with high risk soft tissue sarcoma treated by adjuvant chemotherapy and found small tumor size and low grade as well as location at the extremities to be favourable [18]. Taken together, European clinical practice guidelines encompass chemotherapy as an option in high-risk, deep and large STS (G3; > 5 cm) to be applied either as neoadjuvant or adjuvant therapy only in chemotherapy sensitive histologies [19]. With regard to neo-adjuvant chemotherapy evidence is added by the recent study published by Gronchi et al [20]. In this international phase III study 5 different histologies of high-risk localised sarcoma were randomized to receive either three cycles of histotype-tailored chemotherapy or the standard combination of ifosfamide and doxorubicin before surgery. While it was not possible to show a benefit on disease free survival or overall survival for the histotype-tailored approach the remarkable high projected disease-free survival at 46 months of 62% (95% CI 48–77) for the standard regimen suggests a benefit for neoadjuvant chemotherapy in this situation. Gronchi et al conclude that the added value for the neoadjuvant approach demonstrated in their study may be discussed and incorporated into future guidelines for treatment of high-risk localised sarcoma. However, up to date due to the lack of clear OS benefit in randomized clinical trials, adjuvant chemotherapy is still no general standard but needs to be discussed at an individual basis for each case separately reflecting the risk factors discussed above [21]. Especially the chemo sensitivity of the different histologies needs to be incorporated into this individual benefit-risk assessment [22, 23].

The “sandwich” protocol was originally based on the EORTC 62931 randomized adjuvant trial with some changes. Dose of doxorubicin and ifosfamide per cycle remained the same as in that trial. While radiation therapy in the EORTC 62931 remained optional and was reserved for marginal or R1 resection and 5 cycles of chemotherapy were applied, postoperative radiation in our study was part of the protocol and 4 cycles of chemotherapy were applied.

The completion rate of patients receiving all 5 cycles of chemotherapy at full dose and in time for the EORTC 62391 trial was 68% in comparison to 94% in our cohort. The “sandwich” strategy was chosen in order to apply both postoperative modalities at the earliest possible time point. The hypothesis behind was that this approach might be beneficial in terms of prevention of local as well as distant recurrence. Baseline characteristics of our patients were essentially similar to those from other adjuvant trials in this disease. Further analysis showed that the treatment sequence applied was feasible with manageable toxicity. The acute chemotherapy-related toxicity was mostly transient hematotoxicity, and was found to be of similar extent when compared to other trials in STS, e.g two of the most recent studies published by Davis et al [24] and Hong et al [25].

All but one of the 37 relapses occurred within the first 3 years and was either local or distal. Only one patient died of newly diagnosed and histologically confirmed pulmonary metastases 10 years after the first diagnosis of myxoid liposarcoma. Overall, only 10 out of 104 patients in our study experienced local relapse and all of them occurred within the first 3 years. When comparing our data to the results from the ISG Trial the overall disease-free survival for both arms was 69.7% at 5 years for the whole study population and 68.6% for sarcomas of the extremities [26]. In this study, leiomyosarcoma was the histology with the worst and undifferentiated pleomorphic sarcoma (UPS) the one with the best outcome and this correlated to response rate for chemotherapy at time of resection. The high rate for patients with UPS (41.3% vs 38.4% in the ISG study) and the low rate of leiomyosarcoma (5.8% vs 13.1% in the ISG study) in our study population may at least in part account for the favorable 5-year OS rate of 75.6% (95% CI, 65.2–86.0%) in the whole population and of 79.0% (95% CI, 68.4–89.6%) in the extremities only cohort.

This analysis is not aimed to add evidence for the clinical value of a single part of this trimodality treatment. It rather reflects the results of the long lasting intensive multidisciplinary team approach at our high-volume sarcoma center. Given the limited number of patients in this non-randomized retrospective evaluation, the presented efficacy data should be cautiously interpreted. Also, the relative short median follow up of 39 months forebids any further conclusions and definitely longer follow up is needed. Still, the survival in our patients is among the highest reported and the low local and distant recurrence rate in high-risk STS is at least comparable to the published data.
